# Impaired caudal fin‐fold regeneration in zebrafish deficient for the tumor suppressor Pten

**DOI:** 10.1002/reg2.88

**Published:** 2017-11-10

**Authors:** Alexander James Hale, Ali Kiai, Jelte Sikkens, Jeroen den Hertog

**Affiliations:** ^1^ Hubrecht Institute – KNAW and University Medical Center Utrecht Utrecht The Netherlands; ^2^ Institute Biology Leiden Leiden University Leiden The Netherlands

**Keywords:** PTEN, PTP, regeneration, zebrafish

## Abstract

Zebrafish are able to completely regrow their caudal fin‐folds after amputation. Following injury, wound healing occurs, followed by the formation of a blastema, which produces cells to replace the lost tissue in the final phase of regenerative outgrowth. Here we show that, surprisingly, the phosphatase and tumor suppressor Pten, an antagonist of phosphoinositide‐3‐kinase (PI3K) signaling, is required for zebrafish caudal fin‐fold regeneration. We found that homozygous knock‐out mutant (*ptena^−/−^ptenb^−/−^*) zebrafish embryos, lacking functional Pten, did not regenerate their caudal fin‐folds. AKT phosphorylation was enhanced, which is consistent with the function of Pten. Reexpression of Pten, but not catalytically inactive mutant Pten‐C124S, rescued regeneration, as did pharmacological inhibition of PI3K. Blastema formation, determined by in situ hybridization for the blastema marker *junbb*, appeared normal upon caudal fin‐fold amputation of *ptena^−/−^ptenb^−/−^* zebrafish embryos. Whole‐mount immunohistochemistry using specific markers indicated that proliferation was arrested in embryos lacking functional Pten, and that apoptosis was enhanced. Together, these results suggest a critical role for Pten by limiting PI3K signaling during the regenerative outgrowth phase of zebrafish caudal fin‐fold regeneration.

## 1 INTRODUCTION

Zebrafish (*Danio rerio*) fully regenerate multiple organs after injury, including the heart, retina, spinal cord, and caudal fin, in a process termed epimorphic regeneration (Pfefferli & Jaźwińska, 2015; Poss, Keating, & Nechiporuk, 2003). In broad terms, regeneration of the zebrafish caudal fin proceeds sequentially through three distinct phases: wound healing, blastema formation, and regenerative outgrowth. Adult zebrafish regenerate their caudal fin‐folds within 2 weeks, whilst zebrafish embryos regenerate their caudal fin‐folds within 72 h (Kawakami, Fukazawa, & Takeda, 2004). The mechanism of embryonic caudal fin‐fold regeneration is the same as adult caudal fin regeneration. Following injury, nearby cells migrate to cover the wound and form an apical epidermal cap that is essential to initiate blastema formation and regenerative outgrowth (Poleo, Brown, Laforest, & Akimenko, 2001). To date, many genes have been implicated in the regenerative process (Padhi et al., 2004) and multiple signaling pathways have been validated to be essential for regeneration, including fibroblast growth factor, sonic hedgehog, bone morphogenetic protein, Wnt, and Notch (Gemberling, Bailey, Hyde, & Poss, 2013).

Phosphatase and tensin homologue (PTEN) is one of the most frequently mutated tumor suppressor genes and has a central role in cell signaling. PTEN is a protein‐tyrosine phosphatase and lipid phosphatase with selectivity for the 3‐position of phosphatidylinositol‐(3,4,5)‐trisphosphate (PIP3), and hence PTEN is an antagonist of phosphoinositide‐3‐kinase (PI3K). Activation of PI3K leads to attraction of AKT (also known as PKB) to the plasma membrane and subsequently to phosphorylation of AKT on threonine 308 and serine 473 to activate it (Burgering & Coffer, 1995). The importance of PI3K signaling is highlighted by activated AKT (p‐AKT) interacting with and stimulating many factors presiding over various cellular processes. Notably, p‐AKT promotes cell cycle progression to drive cell proliferation, and enhances cell survival by inhibiting pro‐apoptotic transcription factors (Vara et al., 2004). Elevated p‐AKT has been reported in regenerating caudal fins of medaka (*Oryzias latipes*), and inhibition of PI3K activity using the inhibitor LY294002 inhibits medaka as well as adult caudal fin and embryonic zebrafish caudal fin‐fold regeneration (Nakatani, Nishidate, Fujita, Kawakami, & Kudo, 2008; Rojas‐Muñoz et al., 2009). Inhibition of downstream mechanistic target of rapamycin (mTOR) signaling with rapamycin also inhibits regeneration (Hirose, Shiomi, Hozumi, & Kikuchi, 2014). These studies highlight the importance of PI3K signaling in regeneration. However, the role of PTEN in zebrafish caudal fin regeneration has not been addressed. It is evident that PI3K inhibition attenuates regeneration, and hence one would expect that PTEN inhibition has the opposite effect and enhances regeneration.

To date, the only implication of PTEN in regeneration has been limited to the promotion of central nervous system (CNS) axon regeneration. Various mouse PTEN knockdown or conditional knock‐outs have been generated that show enhanced axon regeneration (Liu et al., 2010; Ohtake, Hayat, & Li, 2015). Also *Caenorhabditis elegans* mutants of *daf‐18*, the PTEN homologue in *C. elegans*, display improved axon regeneration through enhanced mTOR signaling (Byrne et al., 2014). These studies investigated axon regeneration following crushing or axotomy, and suggest that PTEN plays a role in regeneration by limiting regenerative outgrowth of axons. However, axon connections are predominantly rescued by compensatory sprouting from spared neural fibres, and true regenerative outgrowth of injured axons rarely occurs in the adult mouse CNS (He, 2010). Hence, the importance of PTEN in regeneration remains to be determined.

To investigate the role of Pten in the regeneration of whole tissue and to test if *pten* knock‐out mutants have enhanced regeneration, we tested the regenerative capacity of caudal fin‐folds of zebrafish embryos lacking functional Pten (i.e., mutants homozygous for nonsense mutations in both *ptena* and *ptenb*) (Faucherre, Taylor, Overvoorde, Dixon, & den Hertog, 2008). Surprisingly, we found that the caudal fin‐fold of *ptena^−/−^ptenb^−/−^* embryos did not regenerate. AKT phosphorylation was elevated in amputated caudal fin‐folds of *ptena^−/−^ptenb^−/−^* embryos and inhibition of excess PI3K activity restored regeneration. We found evidence that suggests that blastema formation following amputation occurred normally. However, compared to siblings, cell proliferation was arrested and apoptosis enhanced close to the site of amputation.

## 2 RESULTS

### 2.1 Pten catalytic activity is required for zebrafish caudal fin‐fold regeneration

**Figure 1:**
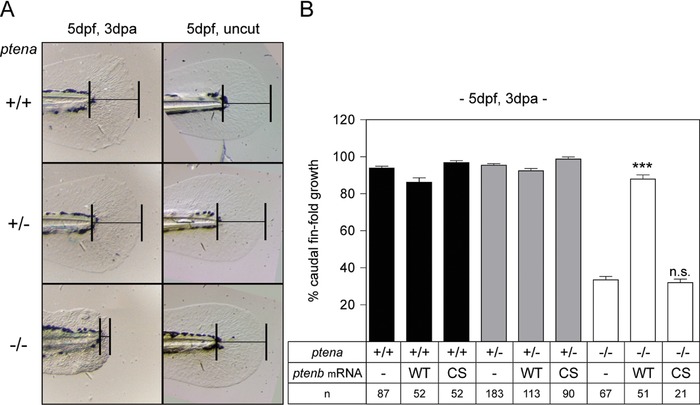
Impaired caudal fin‐fold regeneration in Pten deficient embryos. (A) Embryos from a *ptena^+/−^ptenb^−/−^* in‐cross were micro‐injected at the one‐cell stage with synthetic mRNA encoding WT Ptenb (WT), catalytically inactive Ptenb‐C124S (CS), or were not injected (−). At 2 dpf the caudal fin‐fold was amputated and regeneration was assessed at 3 dpa (i.e., 5 dpf, 3 dpa); equivalent uncut controls were included (i.e., 5 dpf, uncut). All embryos were genotyped. Representative images of non‐injected embryo caudal fin‐folds are shown, and of WT Ptenb or Ptenb‐C124S‐injected embryos in Fig. S1. (B) The means of caudal fin‐fold growth following amputation are depicted relative to caudal fin‐fold growth of uncut *ptena^+/+^ptenb^−/−^* controls. Means of micro‐injected amputated *ptena^−/−^ptenb^−/−^* embryos were compared to non‐injected amputated *ptena^−/−^ptenb^−/−^* embryos. Significance: ^***^ *p *< 0.001; n.s., not significant; error bars indicate standard error of the mean. Data pooled from multiple experiments

To address the role of Pten in regeneration, we first investigated whether zebrafish embryos regenerate their caudal fin‐fold in the absence of functional Pten protein. Zebrafish carry two *pten* genes, *ptena* and *ptenb*, encoding Pten proteins that are functionally redundant (Faucherre et al., 2008). Zebrafish carrying a single wild type (WT) *pten* allele (*ptena^+/−^ptenb^−/−^* or *ptena^−/−^ptenb^+/−^*) show no detectable defects during development or as adults, and they are viable and fertile, but are predisposed to the development of hemangiosarcomas (Choorapoikayil, Kuiper, de Bruin, & den Hertog, 2012). In‐crossing adult *ptena^+/−^ptenb^−/−^* zebrafish produces *ptena^−/−^ptenb^−/−^* embryos and their sibling controls, *ptena^+/−^ptenb^−/−^* and *ptena^+/+^ptenb^−/−^*, that resemble WT embryos for experimentation. Homozygous knock‐out mutant zebrafish embryos (*ptena^−/−^ptenb^−/−^*) develop severe abnormalities during development and are embryonic lethal after 6 days post fertilization (dpf). This is consistent with mouse PTEN knock‐outs that are not viable either (Cristofano et al., 1998). The developmental abnormalities of Pten deficient embryos are apparent from 3 dpf onwards and are characterized by heart edema, curvature and swelling of the body axis, cranial deformations, and vascular hyper‐branching (Choorapoikayil, Weijts, Kers, de Bruin, & den Hertog, 2013). To investigate whether Pten protein is required for caudal fin‐fold regeneration, the caudal fin‐folds of embryos were amputated immediately posterior to the notochord at 2 dpf and allowed to regenerate for 3 days. Representative pictures of regenerating fin‐folds at 3 days post amputation (dpa) and the fin‐folds of uncut controls are shown in Figure 1A. Caudal fin‐fold lengths were determined and are presented as percentage caudal fin‐fold growth, normalized to uncut controls of *ptena^+/+^ptenb^−/−^* embryos (Figs. 1B and S1B). In contrast to their siblings, *ptena^−/−^ptenb^−/−^* embryos displayed severely impaired regeneration. We also observed that the shape of the amputated caudal fin‐fold of *ptena^−/−^ptenb^−/−^* embryos was more rectangular compared to the rounded caudal fin‐folds of their siblings. Fin‐fold growth in uncut controls was indistinguishable between *ptena^−/−^ptenb^−/−^* embryos and siblings (Fig. S1B).

To confirm that the absence of functional Pten is responsible for the lack of regeneration, one‐cell‐stage zebrafish embryos from a *ptena^+/−^ptenb^−/−^* in‐cross were micro‐injected with mRNA encoding either WT Ptenb or catalytically inactive Ptenb‐C124S (Myers et al., 1998). These Ptenb proteins are tagged with C‐terminal mCherry and only positively fluorescent embryos, expressing the Pten‐mCherry fusion proteins, had their caudal fin‐folds amputated and regeneration documented. WT Ptenb rescued caudal fin‐fold regeneration in *ptena^−/−^ptenb^−/−^* embryos, whereas expression of the catalytically inactive Ptenb‐C124S had no effect on caudal fin‐fold regeneration compared to non‐injected controls (−) (Figs. 1 and S1A).

**Figure 2:**
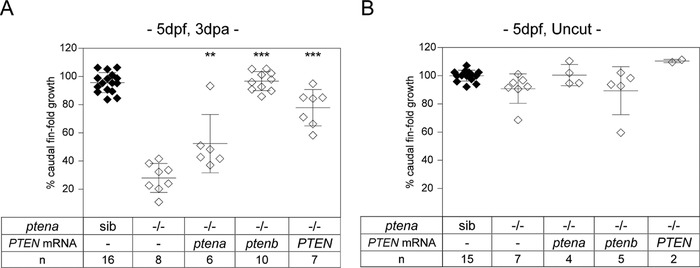
Expression of zebrafish Pten or human PTEN rescues caudal fin‐fold regeneration in Pten deficient embryos. (A) Embryos from a *ptena^+/−^ptenb^−/−^* in‐cross were micro‐injected at the one‐cell stage with synthetic mRNA encoding zebrafish WT Ptena, zebrafish WT Ptenb, or human WT PTEN, or were not injected (−). At 2 dpf the caudal fin‐fold was amputated and regeneration was assessed at 3 dpa (i.e., 5 dpf, 3 dpa); equivalent uncut controls (i.e., 5 dpf, uncut) are shown in (B). All embryos were genotyped. The means of caudal fin‐fold growth following amputation are depicted relative to caudal fin‐fold growth of uncut *ptena^+/+^ptenb^−/−^* controls. Significance: ^***^ *p* < 0.001; ^**^ *p* < 0.01; error bars indicate standard deviation

To test whether Ptena and Ptenb proteins are indeed functionally redundant in caudal fin‐fold regeneration, we repeated the experiment using *ptena^−/−^ptenb^−/−^* embryos obtained from in‐crossing adult *ptena^−/−^ptenb^+/−^* zebrafish. Again, *ptena^−/−^ptenb^−/−^* embryos displayed severely impaired caudal fin‐fold regeneration, and siblings carrying one or two WT *ptenb* alleles regenerated normally, like embryos carrying one or two WT *ptena* alleles (cf. Figs. 1A and S2A). To further show that Ptena and Ptenb are functionally redundant in zebrafish caudal fin‐fold regeneration, *ptena^−/−^ptenb^−/−^* embryos were micro‐injected with mRNA encoding WT Ptena. In addition, as Pten is highly conserved amongst species (Stumpf, Choorapoikayil, & den Hertog, 2015), we tested whether human PTEN could also fulfill the functions of zebrafish Pten in rescuing caudal fin‐fold regeneration by micro‐injecting mRNA encoding human WT PTEN. Both zebrafish Ptena and Ptenb as well as human PTEN rescued caudal fin‐fold regeneration (Fig. 2). In conclusion, Pten catalytic activity is required for regeneration of the zebrafish caudal fin‐fold.

## 3 ELEVATED PI3K SIGNALING BLOCKS CAUDAL FIN‐FOLD REGENERATION IN PTEN DEFICIENT EMBRYOS

**Figure 3:**
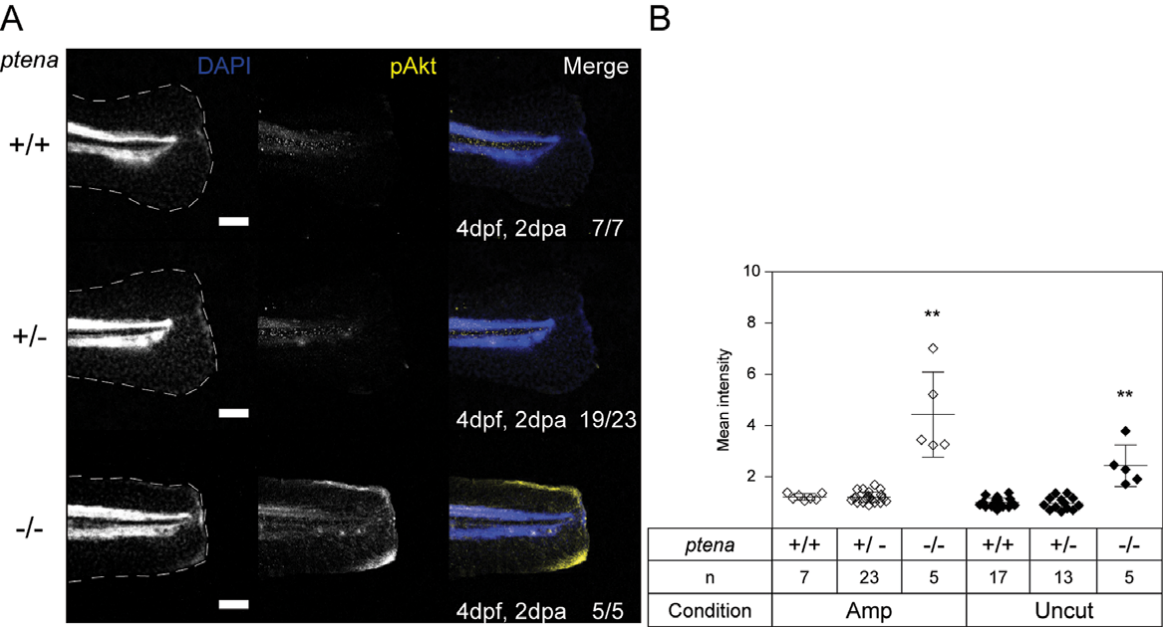
Elevated p‐AKT in the caudal fin‐folds of Pten deficient embryos by 4 dpf. (A) Caudal fin‐folds of embryos from a *ptena^+/−^ptenb^−/−^* in‐cross were amputated and fixed at 2 dpa (i.e., 4 dpf, 2 dpa) and subjected to whole‐mount immunohistochemistry using a p‐AKT‐specific antibody (p‐S473) (yellow). The embryos were counterstained with DAPI (blue). Representative images of amputated embryo caudal fin‐folds are shown and in the left panels the edge of the fin‐fold is indicated with a dashed line. The number of embryos showing similar patterns/total number of embryos analyzed is indicated in the bottom right corner. The scale bar represents 100 μm. (B) p‐AKT was quantified by mean intensity of the region between the notochord and the edge of the caudal fin‐fold. Equivalent uncut controls were also quantified (representative pictures depicted in Fig. S3A). Means within amputated or uncut groups are compared to *ptena^+/+^ptenb^−/−^* embryos. Significance: **p < 0.01; error bars represent standard deviation

PTEN loss, resulting in enhanced PI3K signal transduction, leads to hyper‐active AKT (Stambolic et al., 1998). To confirm that this is also the case in our *ptena^−/−^ptenb^−/−^* embryos, we performed whole‐mount immunohistochemistry for p‐AKT (p‐S473). At both 3 dpf and 4 dpf *ptena^−/−^ptenb^−/−^* embryos displayed elevated p‐AKT levels compared to siblings with a more pronounced effect following fin‐fold amputation (Figs. 3 and S3). Interestingly, elevated p‐AKT levels were particularly apparent at the dorsal and ventral extremities of the zebrafish caudal fin‐fold. This may represent cell‐specific elevation in p‐AKT and should be investigated further.

**Figure 4:**
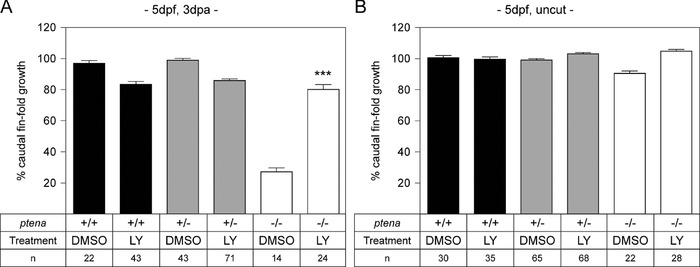
Inhibition of PI3K rescues impaired caudal fin‐fold regeneration in Pten deficient embryos. (A) At 2 dpf the caudal fin‐fold of embryos from a *ptena^+/−^ptenb^−/−^* in‐cross was amputated and incubated with 10 μmol/L LY294002 or 1% dimethyl sulfoxide (DMSO). Regeneration was assessed at 3 dpa (i.e., 5 dpf, 3 dpa). (B) Equivalent uncut controls treated with LY294002 or 1% DMSO from 2 dpf onwards were included (i.e., 5 dpf, uncut). All embryos were genotyped. Regeneration was quantified by measuring the distance from the tip of the notochord to the edge of the caudal fin‐fold. The means of caudal fin‐fold growth are depicted relative to caudal fin‐fold growth of DMSO treated uncut *ptena^+/+^ptenb^−/−^* controls. The mean of LY294002 treated amputated *ptena^−/−^ptenb^−/−^* embryos is compared to DMSO treated amputated *ptena^−/−^ptenb^−/−^* embryos. The number of embryos is indicated (*n*). Significance: ***p < 0.001; error bars indicate standard error of the mean. Data pooled from multiple experiments

We hypothesized that the observed hyper‐activation of AKT in Pten deficient embryos causes impaired regeneration. If this is the case, then restoring p‐AKT levels, by inhibiting over‐active PI3K, should rescue regeneration in Pten deficient mutants. To test this hypothesis, the caudal fin‐folds of zebrafish embryos obtained from a *ptena^+/−^ptenb^−/−^* in‐cross were amputated at 2 dpf and allowed to regenerate for 3 days in the presence or absence of the PI3K inhibitor LY294002. Treatment of *ptena^−/−^ptenb^−/−^* embryos with LY294002 resulted in caudal fin‐fold regeneration comparable with regeneration in siblings (Fig. 4). Uncut controls showed that LY294002 treatment by itself does not have any detrimental effects on caudal fin‐fold growth. These results demonstrate that Pten‐mediated modulation of PI3K activity is required for normal caudal fin‐fold regeneration.

## 4 BLASTEMA FORMATION MARKER SUGGESTS THAT THE INITIAL RESPONSE TO AMPUTATION OF THE CAUDAL FIN‐FOLD IS NORMAL IN ZEBRAFISH EMBRYOS DEFICIENT FOR PTEN

**Figure 5:**
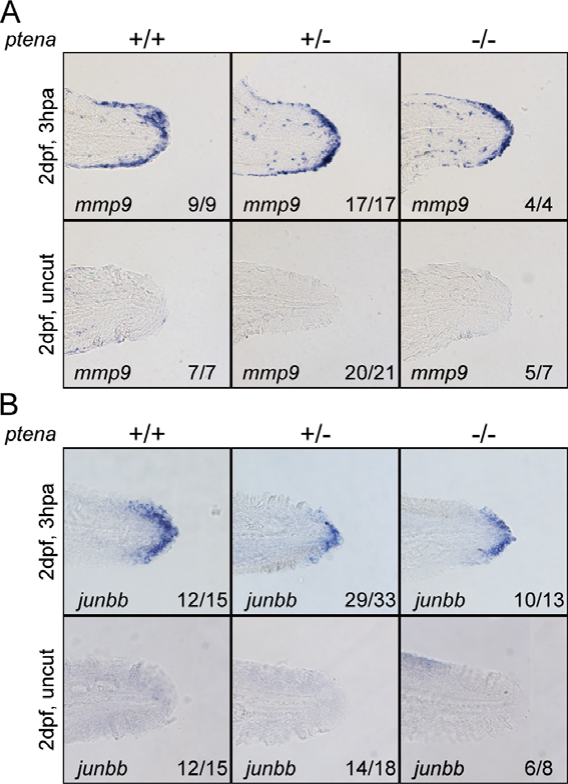
Blastema formation in Pten deficient embryos. At 2 dpf the caudal fin‐fold of embryos from a *ptena^+/−^ptenb^−/−^* in‐cross was amputated and allowed to regenerate. Embryos were fixed at 3 hpa, or equivalent for uncut controls, and subjected to in situ hybridization for *mmp9* (A) or *junbb* (B). Representative images of embryo caudal fin‐folds are shown, and the number of embryos showing similar patterns/total number of embryos analyzed is indicated in the bottom right corner of each panel

We further characterized the regeneration of *ptena^−/−^ptenb^−/−^* mutants by assessing the initial response to amputation, which includes wound healing and formation of the distal blastema. Initially, remaining cells from the dorsal and ventral side of the caudal fin‐fold migrate over the amputation plane to form the wound epithelium (Poleo et al., 2001). This results in the formation of an apical epidermal cap, which signals for the dedifferentiation of cells to form the distal blastema. Amputated zebrafish embryos obtained from a *ptena^+/−^ptenb^−/−^* in‐cross were fixed at 3 h post amputation (hpa) and subjected to in situ hybridization for detection of *mmp9* transcription, which is a marker for the wound epithelium (Yoshinari, Ishida, Kudo, & Kawakami, 2009), and *junbb* and *and1* transcription, both of which have previously been shown to be upregulated in the blastema (Thorimbert et al., 2015; Yoshinari et al., 2009). In fact, *junbb* expression is maintained well into the initial stage of regenerative outgrowth, indicating that *junbb* is a definitive distal blastema marker (Ishida, Nakajima, Kudo, & Kawakami, 2010). Both *mmp9* and *junbb* were clearly induced by amputation of the caudal fin‐fold, and *ptena^−/−^ptenb^−/−^* mutant embryos expressed *mmp9* and *junbb* to a similar extent as their siblings (Fig. 5). There was no obvious difference in *and 1* expression between *ptena^−/−^ptenb^−/−^* embryos and their siblings. However, *and1* expression was already high in uncut controls and was not enhanced in amputated embryos, suggesting that *and1* expression is not a good marker for blastema formation in zebrafish embryos (Fig. S4). Nevertheless, *mmp9* and *junbb* expression suggest that the initial response to amputation occurs normally in Pten deficient embryos.

## 5 ARRESTED PROLIFERATION AND ENHANCED APOPTOSIS IN THE REGENERATING CAUDAL FIN‐FOLD OF PTEN DEFICIENT EMBRYOS

**Figure 6:**
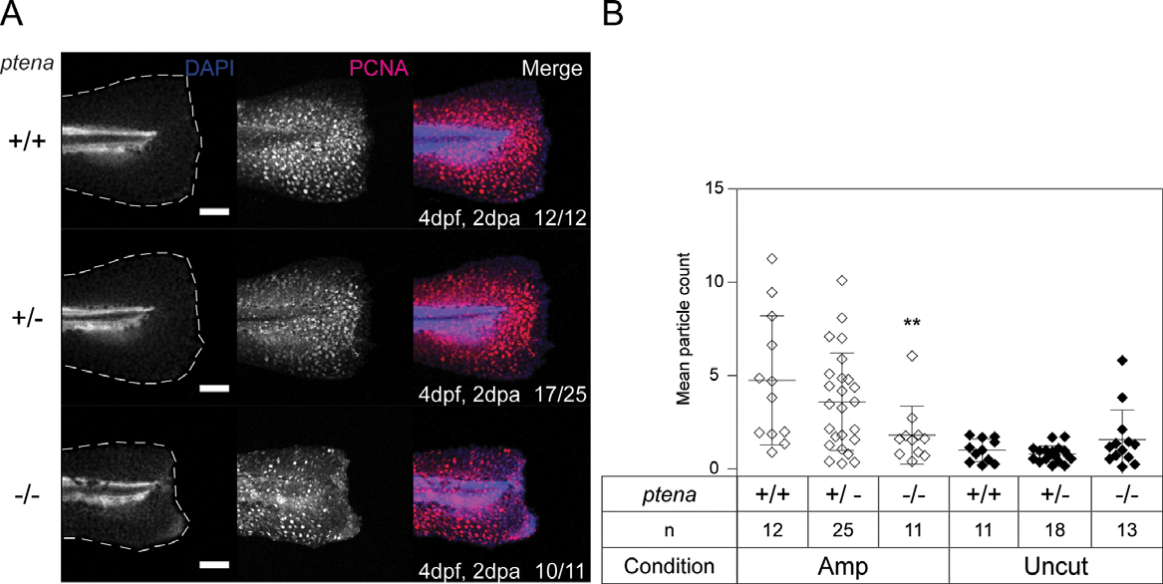
Arrested proliferation at the edge of the amputated caudal fin‐fold of Pten deficient embryos by 2 dpa. (A) Embryos from a *ptena^+/−^ptenb^−/−^* in‐cross were fixed at 2 dpa (i.e., 4 dpf, 2 dpa) and subjected to whole‐mount immunohistochemistry using an antibody specific for PCNA (red). The embryos were counterstained with DAPI (blue). Maximum intensity projection images were taken of the caudal fin‐folds and all embryos were genotyped. Representative images of amputated embryo caudal fin‐folds are shown and the number of embryos showing similar patterns/total number of embryos analyzed is indicated in the bottom right corner of the right panels. The scale bar represents 100 μm. (B) PCNA immunofluorescence between the tip of the notochord and edge of the caudal fin‐fold was quantified by mean particle count. Means within amputated or uncut groups were compared to *ptena^+/+^ptenb^−/−^* embryos. Significance: ^**^ *p *< 0.01; error bars represent standard deviation

Next, we characterized the regeneration of *ptena^−/−^ptenb^−/−^* mutants by assessing proliferation and apoptosis during the regenerative outgrowth stage of caudal fin‐fold regeneration. Proliferation is upregulated during regenerative outgrowth to generate the cells required to form and replace the lost tissue (Kawakami et al., 2004; Poss et al., 2003), and apoptosis is also enhanced and may be required for successful regeneration to proceed (Gauron et al., 2013). Considering that PI3K signaling promotes cell proliferation and enhances cell survival, we analyzed both during the regenerative outgrowth stage. Zebrafish embryos obtained from a *ptena^+/−^ptenb^−/−^* in‐cross were fixed at 1 dpa or 2 dpa and subjected to whole‐mount immunohistochemistry for detection of proliferating cell nuclear antigen (PCNA) expression. PCNA immunofluorescence was high in amputated caudal fin‐folds of siblings (Fig. 6), but remained low in uncut controls (Fig. S5A, B). At 1 dpa PCNA immunofluorescence was found concentrated between the amputation plane and the wound margin at the edge of the caudal fin‐fold, and there was no apparent difference between Pten deficient embryos and their siblings (Fig. S5C, D). By 2 dpa, however, PCNA immunofluorescence was dispersed at the edge of the amputated caudal fin‐fold of *ptena^−/−^ptenb^−/−^* embryos, whereas in siblings that regenerated normally PCNA immunofluorescence remained concentrated between the amputation plane and the wound margin (Fig. 6). Although there was evidence that uncut *ptena^−/−^ptenb^−/−^* embryos have reduced proliferation at 1 dpa compared to *ptena^+/+^ptenb^−/−^* siblings (Fig. S5B, D), this was not the case between *ptena^−/−^ptenb^−/−^* embryos and *ptena^+/−^ptenb^−/−^* siblings, and by 2 dpa this difference was no longer apparent (Figs. S5A, 6B). Together, these results suggest that proliferation was severely impaired during regenerative outgrowth in *ptena^−/−^ptenb^−/−^* embryos at 2 dpa.

**Figure 7:**
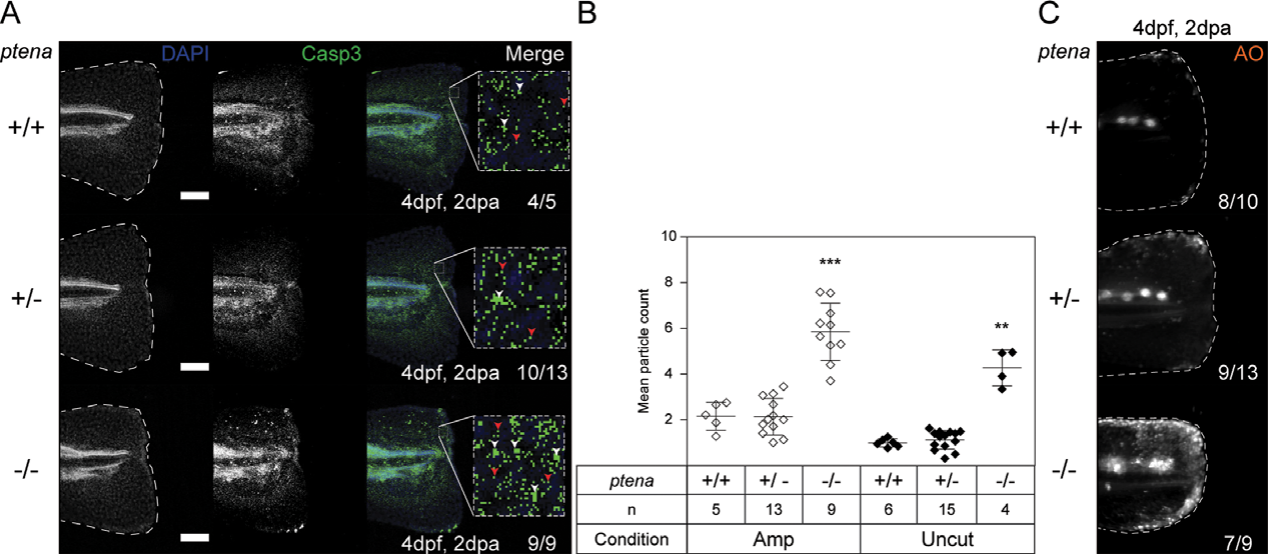
Enhanced apoptosis in the caudal fin‐folds of Pten deficient embryos by 4 dpf. (A) Embryos from a *ptena^+/−^ptenb^−/−^* in‐cross were amputated at 2 dpf, fixed at 2 dpa and subjected to whole‐mount immunohistochemistry using an antibody specific for activated caspase‐3 (green). Embryos were counterstained with DAPI (blue). Representative images of amputated embryo caudal fin‐folds are shown, and the number of embryos showing similar patterns/total is indicated. Scale bar: 100 μm. Zoomed images of each caudal fin‐fold are shown. White arrowheads indicate cells; red arrowheads indicate background staining. (B) Caspase‐3 immunofluorescence was quantified by mean particle count of the caudal fin‐fold, following thresholding and size restriction to reduce background signal. Means within amputated or uncut groups were compared to *ptena^+/+^ptenb^−/−^* embryos. Significance: ^**^ *p *< 0.01; ^***^ *p *< 0.001; error bars represent standard deviation. (C) Embryos from a *ptena^+/−^ptenb^−/−^* in‐cross were amputated and allowed to regenerate. Live embryos were stained with acridine orange (AO), for 30 min at 2 dpa (i.e., 4 dpf, 2 dpa). Representative images of embryo caudal fins are shown. AO staining at 3 dpf and of equivalent uncut controls at 4 dpf is shown in Fig. S7

We used whole‐mount immunohistochemistry with an activated caspase‐3‐specific antibody to assess apoptosis. At both 1 dpa and at 2 dpa activated caspase‐3 is enhanced in *ptena^−/−^ptenb^−/−^* embryos compared to siblings (Figs. S6, 7A, B). Representative images of caudal fin‐folds of live embryos stained with acridine orange, for cells undergoing apoptosis (Tucker & Lardelli, 2007), confirmed that *ptena^−/−^ptenb^−/−^* embryos experienced enhanced apoptosis compared to siblings (Figs. 7C, S7). Taken together, the lack of regeneration in *ptena^−/−^ptenb^−/−^* embryos is probably due to arrested proliferation and enhanced apoptosis.

## 6 DISCUSSION

Our results demonstrate a critical role for Pten in zebrafish caudal fin‐fold regeneration. Zebrafish embryos lacking functional Pten (*ptena^−/−^ptenb^−/−^*) do not regenerate their caudal fin‐folds following amputation (Fig. 1). We propose that the increased p‐AKT levels in *ptena^−/−^ptenb^−/−^* embryos (Figs. 3 and S3) inhibit caudal fin‐fold regeneration, which is consistent with our experiments to restore caudal fin‐fold regeneration by balancing the increased PI3K signaling, by either re‐expressing WT Ptenb (Figs. 1B and S1A) or inhibiting excess PI3K activity with LY294002 (Fig. 4). Although we cannot exclude that the inability of *ptena^−/−^ptenb^−/−^* embryos to regenerate their caudal fin‐folds is secondary to developmental abnormalities, the lack of caudal fin‐fold regeneration at 1 dpa (3 dpf), when no morphological abnormalities are present, suggests that this is unlikely. Furthermore, the rescue of caudal fin‐fold regeneration by treatment with LY294002, specifically during regenerative outgrowth, strongly suggests that Pten has a specific role during caudal fin‐fold regeneration.

Normally, fin amputation induces three sequential steps to replace the lost fin: wound healing, blastema formation, and regenerative outgrowth. Following wound healing, an apical epidermal cap is produced that signals for the formation of the blastema, and thus successful blastema formation is indicative of successful wound healing. The expression of *mmp9* (Fig. 5) in embryos deficient for Pten suggests that wound healing occurs normally. Furthermore, we show that the amputated caudal fin‐folds of *ptena^−/−^ptenb^−/−^* embryos express *junbb*, like their siblings (Fig. 5), suggesting that both wound healing and formation of the distal blastema occur normally in the absence of Pten. Whereas inhibition of PI3K with LY294002 has been shown to prevent blastema formation (Nakatani et al., 2008), our results suggest that excess PI3K activity is not detrimental to either wound healing or formation of the distal blastema.

Regenerative outgrowth is characterized by proliferation and differentiation of cells to replace the lost tissue. Whilst cells of amputated *ptena^−/−^ptenb^−/−^* embryo caudal fin‐folds initially proliferate as normal (Fig. S5C), by 2 dpa proliferation is arrested compared to siblings (Fig. 6). This result is surprising as it is well demonstrated that PTEN loss leads to increased proliferation (Shaw & Cantley, 2006). However, this response occurs in tumor cells, whereas the cells of developing *ptena^−/−^ptenb^−/−^* embryos are otherwise normal. Oncogenes expressed in normal healthy cells induce senescence (Gorgoulis & Halazonetis, 2010), and hyper‐active AKT has been shown to do the same in mouse embryonic and human fibroblasts (Astle et al., 2012; Chen et al., 2005). Moreover, T‐cell senescence occurs in some immunodeficient patients with germline gain‐of‐function mutations in PI3K (Lucas et al., 2014). Therefore, elevated p‐AKT in our *ptena^−/−^ptenb^−/−^* embryos (Figs. 3 and S3) may induce senescence that results in proliferation arrest by 2 dpa.

Activated caspase‐3 positive cells are increased in caudal fin‐folds of *ptena^−/−^ptenb^−/−^* embryos compared to siblings (Figs. 6, S6 and S7), which is surprising because loss of PTEN is often associated with suppression of apoptosis and enhanced cell survival (Brunet et al., 1999; Kulik, Klippel, & Weber, 1997). These responses are elicited in immortalized cell lines or cell lines following irradiation‐induced DNA damage, which are not normal, WT cells or conditions. Hyper‐active AKT in normal human cells initiates the release of pro‐inflammatory cytokines, a hallmark of senescence‐associated secretory phenotype (Astle et al., 2012), which is thought to induce apoptosis of senescent cells (Freund, Orjalo, Desprez, & Campisi, 2010). Thus, the elevated p‐AKT in *ptena^−/−^ptenb^−/−^* embryos may induce senescence and apoptosis. However, one would expect apoptosis to follow arrested proliferation in AKT‐induced senescence, whilst in *ptena^−/−^ptenb^−/−^* embryos enhanced apoptosis precedes arrested proliferation (cf. Figs. 7 and S6 with Figs. 6 and S5).

In addition, arrested proliferation was not observed in the uncut caudal fin‐folds of *ptena^−/−^ptenb^−/−^* embryos (Figs. S5A and 6B), suggesting that elevated p‐AKT alone is not sufficient to induce senescence and that caudal fin‐fold amputation has an additional effect that leads to arrested proliferation in amputated *ptena^−/−^ptenb^−/−^* embryos. In *Rxra* knock‐out mouse embryos, where gastrulation defects during embryogenesis lead to cardiac defects and ultimately lethality by E15.5, apoptosis precedes reduced proliferation (Kubalak, Hutson, Scott, & Shannon, 2002). That study attributed this to upregulated transforming growth factor β (TGFβ) signaling. Identifying the consequences of *pten* knock‐out in other signaling pathways implicated in caudal fin‐fold regeneration may explain why apoptosis is enhanced and proliferation becomes arrested by 2 dpa.

In conclusion, we demonstrate for the first time that Pten is required for embryonic zebrafish caudal fin‐fold regeneration. Loss of Pten inhibits caudal fin‐fold regeneration associated with reduced proliferation, enhanced apoptosis, and elevated AKT activation. Restoring Pten expression or inhibiting PI3K activity during regenerative outgrowth rescues regeneration in embryos lacking endogenous Pten protein. Pten probably functions to balance PI3K signaling to coordinate proper proliferation and apoptosis during regenerative outgrowth.

## 7 MATERIALS AND METHODS

### 7.1 Zebrafish husbandry

All procedures involving experimental animals were approved by the local animal experiments committee (Koninklijke Nederlandse Akademie van Weterschappen − Dierexperimenten commissie KNAW‐DEC protocol HI12.0701) and performed according to local guidelines and policies in compliance with national and European law. The *ptena^+/−^ptenb^−/−^* and *ptena^−/−^ptenb^+/−^* zebrafish lines in the Tuebingen Long fin (TL) background were previously created by target‐selected gene inactivation (TSGI), and both *ptena^hu1864^* and *ptenb^hu1435^* alleles result from nonsense mutations that lead to a premature stop codon upstream of the catalytic cysteine, Cys124 (Faucherre et al., 2008). Adult *ptena^+/−^ptenb^−/−^* and *ptena^−/−^ptenb^+/−^* zebrafish were in‐crossed to generate *ptena^−/−^ptenb^−/−^* embryos for all experiments. Zebrafish were raised and maintained as described by Westerfield (2000) under a 14 h light/10 h dark cycle at 28.5°C.

### 7.2 mRNA synthesis and micro‐injections

The constructs pCS2+‐zfPtenb‐mCherry, pCS2+‐zfPtena‐mCherry, pCS2+‐hPTEN‐mCherry, and pCS2+‐zfPtenb‐C124S‐mCherry were obtained as described previously (Faucherre et al., 2008). Sense messenger RNA (mRNA) synthesis and micro‐injection into one‐cell‐stage zebrafish embryos was performed as described previously (Stumpf & Den Hertog, 2016).

### 7.3 Caudal fin‐fold amputation

Zebrafish embryos were amputated as previously described (Hale & den Hertog, 2016); amputations were performed at 2 dpf for all experiments. Regeneration was allowed to proceed until analysis at 3 dpa or fixation at either 1 dpa or 2 dpa. LY294002 hydrochloride (Sigma) was administered directly following recovery of amputated embryos in E3 medium. Whole zebrafish embryos were lysed for genotyping or fixed in 4% paraformaldehyde (PFA), in phosphate‐buffered saline (PBS), either 3 hpa for in situ hybridization or at 1 dpa and 2 dpa for immunohistochemistry.

### 7.4 *In situ* hybridization

*In situ* hybridizations were performed as previously described (Thisse, Thisse, Schilling, & Postlethwait, 1993), using *mmp9*, *and1*, and *junbb* digoxigenin‐UTP‐labeled anti‐sense riboprobes. Parts of *mmp9*, *and1*, and *junbb* were amplified from zebrafish cDNA using specific primers (Table S1) and the resulting polymerase chain reaction (PCR) products were cloned into vectors and used as DNA templates for synthesis of riboprobes. Digoxigenin‐UTP‐labeled anti‐sense riboprobes were synthesized from *and1* plasmid DNA, or *mmp9* or *junbb* PCR product with a T7 promoter, produced using the primers in Table S1. Caudal fin‐folds of embryos were severed and mounted in 70% glycerol in PBS for imaging on a Zeiss Axioskop 2 Mot Plus microscope with either a Plan‐NEOFLUAR 10×/0.30 or 20×/0.50 objective. The rest of the embryo was lysed for genotyping.

### 7.5 Immunohistochemistry

Zebrafish embryos fixed in 4% PFA were washed in PBS 0.1% Tween‐20 and antigen retrieval was performed depending on the antibody used: ice‐cold acetone for 20 min for PCNA; ice‐cold methanol for 20 min for activated caspase‐3 (Sorrells, Toruno, Stewart, & Jette, 2013); and 10 mmol/L Tris, 1 mmol/L ethylenediaminetetraacetic acid, pH 9.0, for p‐AKT (Ser473) (van der Velden et al., 2011). Whole embryos were incubated overnight at 4°C in mouse anti‐PCNA (1:200, #M0879; DAKO Agilent Pathology Solutions), rabbit anti‐activated caspase‐3 (1:200, #9661; Cell Signaling Technology), or rabbit anti‐p‐AKT (Ser473) (1:50; #4060; Cell Signaling Technology). Secondary antibodies conjugated to Cy5 goat anti‐mouse or goat anti‐rabbit IgG were used 1:500 and 1:200 respectively (#115‐175‐146 and #111‐175‐144; Jackson ImmunoResearch). Nuclei were shown by DAPI (4′,6‐diamidino‐2‐phenylindole) staining. Caudal fin‐folds of embryos were mounted for imaging in 70% glycerol in PBS, and the rest of the embryo was lysed for genotyping. *Z*‐stacks (6 μm step size) of the caudal fin‐fold were acquired for every embryo with a Leica Sp8 confocal microscope. ImageJ software (https://rsb.info.nih.gov./ij/) was used to generate maximum intensity (*z*) projections and merge channels. Quantification was performed on the original *z*‐projections (1024 × 1024 px, 300 dpi). PCNA immunostaining between the tip of the notochord and edge of the caudal fin‐fold was quantified by cropping the *z*‐projections to remove the signal adjacent to the tip of the notochord and applying rolling ball background subtraction with an average from 5 px. Particles were counted for PCNA immunofluorescence following a black and white thresholding of 40−255, applying watershed, and using a size restriction of 0.00009 inch^2^−infinity. Activated caspase‐3 immunostaining was quantified following rolling ball background subtraction with an average from 50 px. Particles were counted for activated caspase‐3 following a black and white thresholding of 121−255 and using a size restriction of 0.000009−0.000144 inch^2^. The mean intensity of p‐AKT was measured from the wound margin inwards using a region of interest with dimensions equivalent to height 1.58 μm (475 px) and width 0.78 μm (235 px) for uncut embryos and width 0.45 μm (135 px) for amputated embryos.

### 7.6 Acridine orange staining

Zebrafish embryos were placed in acridine orange staining solution diluted in E3 medium in the dark for 30 min at 28.5°C. They were washed once with E3 medium and anesthetized in 0.1% MS222. Images of zebrafish embryo caudal fin‐folds were acquired with a Leica M165 FC microscope using a Leica EL6000 fluorescence lamp, GFP filter, and Leica DFC420 C camera.

### 7.7 Genotyping

All embryos that were used in these assays were genotyped to establish *ptena* and/or *ptenb* status. To this end, genomic zebrafish DNA was extracted through lysis of embryos in 100 μg/mL proteinase K (Sigma) diluted in SZL buffer (50 mmol/L KCl, 2.5 mmol/L MgCl, 10 mmol/L Tris pH 8.3, 0.005% NP40, 0.005% Tween‐20, and 10% 0.1% gelatine). Lysis was performed by incubating at 60°C for 1 h, followed by 95°C for 15 min in a thermal cycler (BioRad T100). Primers of *ptena* and *ptenb*, containing the nonsense mutations of the *ptena^hu1864^* and *ptenb^hu1435^* alleles (Table S1), were mixed with genomic zebrafish DNA and Kompetitive Allele Specific PCR (KASP) master mix (LGC Group). Amplification was carried out according to the manufacturer's instructions and the resulting PCR products were analyzed in a PHERAstar microplate reader (BMG LABTECH). Klustercaller software was used to identify the mutations.

### 7.8 Statistics

For analysis of caudal fin‐fold lengths, histograms of whole datasets were examined to determine non‐normal distribution of the data. Statistical analysis of unequal variances was obtained through a Kruskal−Wallis test. Differences between different experimental conditions were assessed for significance using a Mann−Whitney *U* test. Differences were considered significant for *p *< 0.01, and if they satisfied a confidence interval of 99% in a Monte Carlo exact test. All tests for regenerating caudal fin‐folds were performed in SPSS (IBM). For analysis of immunohistochemistry measurements, differences between different experimental conditions were assessed for significance using a Mann−Whitney *U* test with a confidence level set to 95%. All tests for immunohistochemistry measurements were performed in GraphPad Prism (GraphPad Software). Differences were considered significant for *p *< 0.05.

## CONFLICT OF INTEREST

No conflict of interest to declare.

## Supporting information

Table S1. Primers used for amplifying *mmp9*, *and1*, and *junbb* from zebrafish embryo cDNA, and *ptena* and *ptenb* from genomic zebrafish DNA.
Fig. S1. Impaired caudal fin‐fold regeneration in Pten deficient embryos. (A) Embryos from a *ptena^+/−^ptenb^−/−^* in‐cross were micro‐injected at the one‐cell stage with synthetic mRNA encoding WT Ptenb (WT), catalytically inactive Ptenb‐C124S (CS), or were not injected (−). At 2 dpf the caudal fin‐fold was amputated and regeneration was assessed after 3 days (i.e., 5 dpf, 3 dpa); equivalent uncut controls were included (i.e., 5 dpf, uncut). All embryos were genotyped. Representative images of micro‐injected embryo caudal fin‐folds are shown. (B) Uncut caudal fin‐fold growth was quantified by measuring the distance from the tip of the notochord to the edge of the caudal fin‐fold. The means of uncut caudal fin‐fold growth are depicted relative to caudal fin‐fold growth of uncut *ptena^+/+^ptenb^−/−^* controls. Means of micro‐injected *ptena^−/−^ptenb^−/−^* embryos were compared to non‐injected *ptena* ^−/−^ *ptenb^−/−^* embryos. Error bars indicate standard error of the mean.
Fig. S2. Impaired caudal fin‐fold regeneration in Pten deficient embryos. (A) Embryos from a *ptena^−/−^ptenb^+/−^* in‐cross were micro‐injected at the one‐cell stage with synthetic mRNA encoding WT Ptenb (WT), catalytically inactive Ptenb‐C124S (CS), or were not injected (−). At 2 dpf the caudal fin‐fold was amputated and regeneration was assessed after 3 days (i.e., 5 dpf, 3 dpa). (B) Equivalent uncut controls were included (i.e., 5 dpf, uncut). All embryos were genotyped. The means of caudal fin‐fold growth are depicted relative to caudal fin‐fold growth of uncut *ptena^−/−^ptenb^+/+^* controls. Means of micro‐injected *ptena^−/−^ptenb^−/−^* embryos were compared to non‐injected *ptena^−/−^ptenb^−/−^* embryos. Error bars indicate standard error of the mean.
Fig. S3. Elevated p‐AKT in the caudal fin‐folds of Pten deficient embryos. (A) Uncut embryos from a *ptena^+/−^ptenb^−/−^* in‐cross were fixed at 4 dpf (4 dpf, uncut) in parallel to the embryos depicted in Fig. 2A. (B), (C) Embryos from a *ptena^+/−^ptenb^−/−^* in‐cross were amputated and fixed at 1 dpa (i.e., 3 dpf, 1 dpa); equivalent uncut controls (3 dpf, uncut) were included. Embryos were subjected to whole‐mount immunohistochemistry using a p‐AKT‐specific antibody (p‐S473) (yellow). The embryos were counterstained with DAPI (blue). Representative images of embryo caudal fin‐folds are shown, and in the left panels the edge of the fin‐fold is indicated with a dashed line. The number of embryos showing similar patterns/total number of embryos analyzed is indicated in the bottom right corner. The scale bar represents 100 μm. (D) p‐AKT immunofluorescence was quantified by mean intensity of the region between the notochord and the edge of the caudal fin‐fold. Means within amputated or uncut groups are compared to *ptena^+/+^ptenb^−/−^* embryos. The number of embryos analyzed is indicated (*n*). Significance: ***p < 0.001; error bars represent standard deviation. Quantification of p‐AKT immunofluorescence at 4 dpf is depicted in Fig. 2B.
Fig. S4. In situ hybridization staining of *and1* in Pten deficient embryos. At 2 dpf the caudal fin‐fold of embryos from a *ptena^+/−^ptenb^−/−^* in‐cross was amputated and allowed to regenerate. Embryos were fixed at 3 hpa, or equivalent for uncut controls, and subjected to in situ hybridization for *and1*. Representative images of embryo caudal fin‐folds are shown with the number of embryos/total number of embryos in the bottom right corner of each panel.
Fig. S5. Normal proliferation in the caudal fin‐folds of Pten deficient embryos. (A) Uncut embryos from a *ptena^+/−^ptenb^−/−^* in‐cross were fixed at 4 dpf (4 dpf, uncut) in parallel to the embryos depicted in Fig. 5. (B), (C) Embryos from a *ptena^+/−^ptenb^−/−^* in‐cross were amputated and fixed at 1 dpa (i.e., 3 dpf, 1 dpa); equivalent uncut controls (3 dpf, uncut) were included. Embryos were subjected to whole‐mount immunohistochemistry using an antibody specific for the cell proliferation marker PCNA (red). The embryos were counterstained with DAPI (blue). Representative images of embryo caudal fin‐folds are shown, and in the left panels the edge of the fin‐fold is indicated with a dashed line. The number of embryos showing similar patterns/total number of embryos analyzed is indicated in the bottom right corner. The scale bar represents 100 μm. (D) PCNA immunofluorescence between the tip of the notochord and edge of the caudal fin‐fold at 3 dpf was quantified by mean particle count, with thresholding and size restriction to remove background signal. Equivalent uncut controls were also quantified. Means within amputated or uncut groups were compared to *ptena^+/+^ptenb^−/−^* embryos. Significance: *p < 0.05; error bars represent standard deviation. Quantification of PCNA immunofluorescence at 4 dpf is depicted in Fig. 6B.
Fig. S6. Enhanced apoptosis in the caudal fin‐folds of Pten deficient embryos. (A) Uncut embryos from a *ptena^+/−^ptenb^−/−^* in‐cross were fixed at 4 dpf (4 dpf, uncut) in parallel to the embryos depicted in Fig. 6A. (B), (C) Embryos from a *ptena^+/−^ptenb^−/−^* in‐cross were amputated and fixed at 1 dpa (i.e., 3 dpf, 1 dpa); equivalent uncut controls (3 dpf, uncut) were included. Embryos were subjected to whole‐mount immunohistochemistry using an antibody specific for the apoptosis marker activated caspase‐3 (green). The embryos were counterstained with DAPI (blue). Representative images of embryo caudal fin‐folds are shown, and in the left panels the edge of the fin‐fold is indicated with a dashed line. The number of embryos showing similar patterns/total number of embryos analyzed is indicated in the bottom right corner. The scale bar represents 100 μm. Zoomed images of each caudal fin‐fold are shown. White arrowheads indicate cells; red arrowheads indicate background staining. (D) Caspase‐3 immunofluorescence was quantified by mean particle count of the caudal fin‐fold following thresholding and size restriction to reduce background signal. Means within amputated or uncut groups are compared to *ptena^+/+^ptenb^−/−^* embryos. The number of embryos analyzed is indicated (*n*). Significance: *p < 0.05; **p < 0.01; error bars represent standard deviation. Quantification of caspase‐3 immunofluorescence at 4 dpf is depicted in Fig. 6B.
Fig. S7. Enhanced apoptosis in the caudal fin‐folds of live Pten deficient embryos. Embryos from a *ptena^+/−^ptenb^−/−^* in‐cross were amputated and allowed to regenerate. Embryos were stained at 2 dpa (i.e., 4 dpf, 2 dpa) or 1 dpa (i.e., 3 dpf, 1 dpa), or equivalent for uncut controls (i.e., 4 dpf, uncut; or 3 dpf, uncut), with a dye for cells undergoing apoptosis, acridine orange, for 30 min. Representative images of embryo caudal fin‐folds are shown, and the edge of the fin‐fold is indicated with a dashed line. The number of embryos showing similar patterns/total number of embryos analyzed is indicated in the bottom right corner. Representative images for embryos stained at 4 dpf, 2 dpa are shown in Fig. 7C.
