# Supplementary material for: Impaired caudal fin‐fold regeneration in zebrafish deficient for the tumor suppressor Pten
Source: Regeneration (Oxf). 2017 Nov 10;4(4):217–26. doi: 10.1002/reg2.88 (PMC5743786; doi:10.1002/reg2.88)
Supplement: Supplementary file 1 — Table S1. Primers used for amplifying mmp9, and1, and junbb from zebrafish embryo cDNA, and ptena and ptenb from genomic zebrafish DNA. Fig. S1. Impaired caudal fin‐fold regeneration in Pten deficient embryos. (A) Embryos from a ptena+/−ptenb−/− in‐cross were micro‐injected at the one‐cell stage with synthetic mRNA encoding WT Ptenb (WT), catalytically inactive Ptenb‐C124S (CS), or were not injected (−). At 2 dpf the caudal fin‐fold was amputated and regeneration was assessed after 3 days (i.e., 5 dpf, 3 dpa); equivalent uncut controls were included (i.e., 5 dpf, uncut). All embryos were genotyped. Representative images of micro‐injected embryo caudal fin‐folds are shown. (B) Uncut caudal fin‐fold growth was quantified by measuring the distance from the tip of the notochord to the edge of the caudal fin‐fold. The means of uncut caudal fin‐fold growth are depicted relative to caudal fin‐fold growth of uncut ptena+/+ptenb−/− controls. Means of micro‐injected ptena−/−ptenb−/− embryos were compared to non‐injected ptena −/− ptenb−/− embryos. Error bars indicate standard error of the mean. Fig. S2. Impaired caudal fin‐fold regeneration in Pten deficient embryos. (A) Embryos from a ptena−/−ptenb+/− in‐cross were micro‐injected at the one‐cell stage with synthetic mRNA encoding WT Ptenb (WT), catalytically inactive Ptenb‐C124S (CS), or were not injected (−). At 2 dpf the caudal fin‐fold was amputated and regeneration was assessed after 3 days (i.e., 5 dpf, 3 dpa). (B) Equivalent uncut controls were included (i.e., 5 dpf, uncut). All embryos were genotyped. The means of caudal fin‐fold growth are depicted relative to caudal fin‐fold growth of uncut ptena−/−ptenb+/+ controls. Means of micro‐injected ptena−/−ptenb−/− embryos were compared to non‐injected ptena−/−ptenb−/− embryos. Error bars indicate standard error of the mean. Fig. S3. Elevated p‐AKT in the caudal fin‐folds of Pten deficient embryos. (A) Uncut embryos from a ptena+/−ptenb−/− in‐cross were fixed at 4 dpf (4 dp [file REG2-4-217-s001.pdf]

Supporting Information

Table S1. Primers used for amplifying *mmp9*, *and1*, and *junbb* from zebrafish embryo cDNA, and *ptena* and *ptenb* from genomic zebrafish DNA.

Primer	Sequence
mmp9 FWD 1	TCCTGGAGATGTGATCAAGAA
mmp9 REV 1	GGCCCTCACTGGTGCAGGATG
mmp9 FWD 2	CACAGCTAGCGGATGAGTATCTGAAGC
mmp9-T7 REV 2	TAATACGACTCACTATAGAATGGAAAATGGCATGGCTCTCC
and1 FWD	CAAGACAGGCCTTGAGGAAG
and1-T7 REV	TAATACGACTCACTATAGTTGGGAACTTAGTGGGATGC
junbb FWD 1	TGGGTTACGGTCACAACGAC
junbb REV 1	CAGTGTCCGTTCTCTTCCGT
BamHI-*junbb* FWD (nested 1)	ATAGGATCCTACACGACGCTGAACGCATA
junbb-EcoRI REV (nested 2)	CTCGAATTCGTGTCCGTTCTCTTCCGTCC
junbb FWD 2	TACACGACGCTGAACGCATA
junbb-T7 REV 2	TAATACGACTCACTATAGGTGTCCGTTCTCTTCCGTCC
ptena WT FWD	GAAGGTGACCAAGTTCATGCTTGGGCTTTCCAGCCGAAC
ptena KO FWD	GAAGGTCGGAGTCAACGGATTTGGGCTTTCCAGCCGAAT
ptena REV	TCAATGTTGTTTCGGTAAACACCTTCCAA
ptenb WT FWD	GAAGGTGACCAAGTTCATGCTGTTTCTTGATTCAAAGCATAAAGATCATTAC
ptenb KO FWD	GAAGGTCGGAGTCAACGGATTGTTTCTTGATTCAAAGCATAAAGATCATTAA
ptenb REV	CTGAAGTTTTTGAAACACACTTACAGGTT

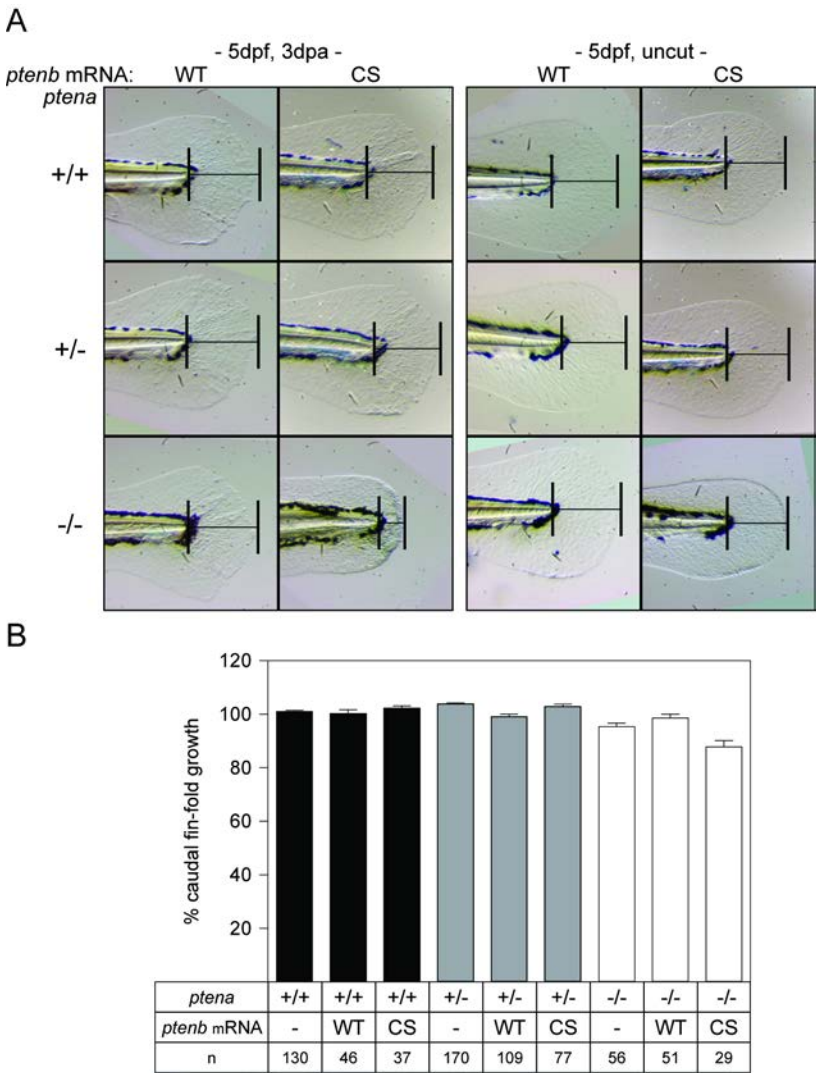

Fig. S1. Impaired caudal fin-fold regeneration in Pten deficient embryos. (A) Embryos from a *ptena*$^{+/-}$ *ptenb*$^{-/-}$ in-cross were micro-injected at the one-cell stage with synthetic mRNA encoding WT Ptenb (WT), catalytically inactive Ptenb-C124S (CS), or were not injected (-). At 2dpf the caudal fin-fold was amputated and regeneration was assessed after 3 days (*i.e.* 5dpf, 3dpa), equivalent uncut controls were included (*i.e.* 5dpf, uncut). All embryos were genotyped. Representative images of micro-injected embryo caudal fin-folds are shown. (B) Uncut caudal fin-fold growth was quantified by measuring the distance from the tip of the notochord to the edge of the caudal fin-fold. The means of uncut caudal fin-fold growth are depicted relative to caudal fin-fold growth of uncut *ptena*$^{+/+}$*ptenb*$^{-/-}$ controls. Means of micro-injected *ptena*$^{-/-}$*ptenb*$^{-/-}$ embryos were compared to non-injected *ptena*$^{-/-}$ *ptenb*$^{-/-}$ embryos. Error bars indicate standard error of the mean.

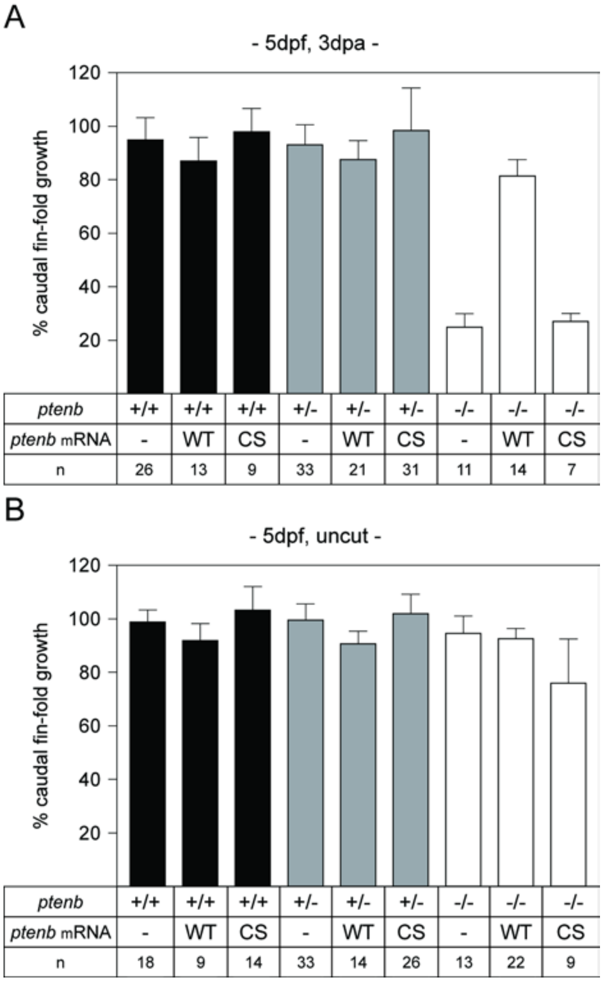

Fig. S2. Impaired caudal fin-fold regeneration in Pten deficient embryos. (A) Embryos from a *ptena*$^{-/-}$ *ptenb*$^{+/-}$ in-cross were micro-injected at the one-cell stage with synthetic mRNA encoding WT Ptenb (WT), catalytically inactive Ptenb-C124S (CS), or were not injected (-). At 2dpf the caudal fin-fold was amputated and regeneration was assessed after 3 days (*i.e.* 5dpf, 3dpa). (B) Equivalent uncut controls were included (*i.e.* 5dpf, uncut). All embryos were genotyped. The means of caudal fin-fold growth are depicted relative to caudal fin-fold growth of uncut *ptena*$^{-/-}$*ptenb*$^{+/+}$ controls. Means of micro-injected *ptena*$^{-/-}$*ptenb*$^{-/-}$ embryos were compared to non-injected *ptena*$^{-/-}$*ptenb*$^{-/-}$ embryos. Error bars indicate standard error of the mean.

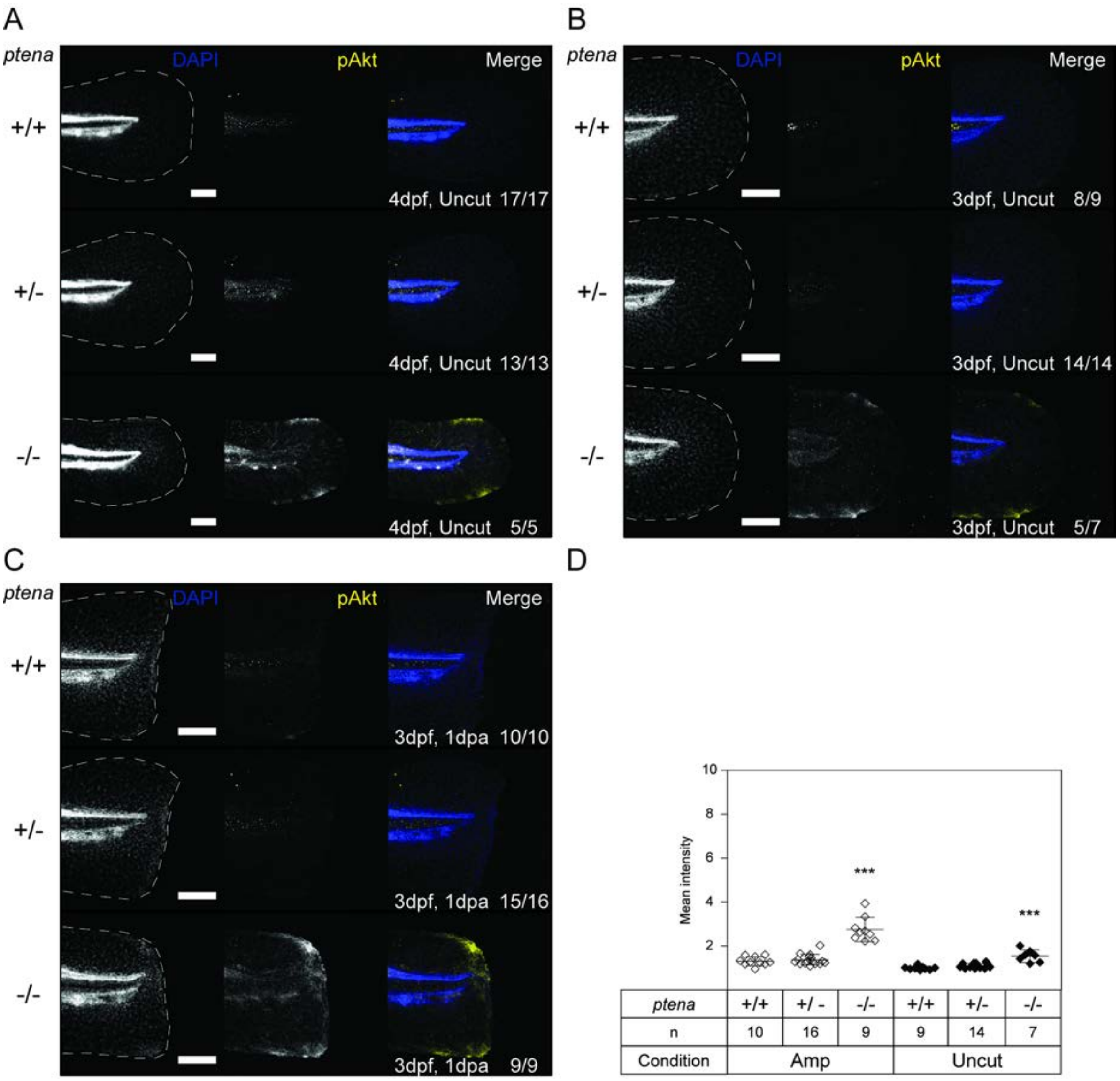

Fig. S3. Elevated p-AKT in the caudal fin-folds of Pten deficient embryos. (A) Uncut embryos from a *ptena*$^{+/-}$*ptenb*$^{-/-}$ in-cross were fixed at 4dpf (4dpf, uncut) in parallel to the embryos depicted in Fig. 2A. (B, C) Embryos from a *ptena*$^{+/-}$*ptenb*$^{-/-}$ in-cross were amputated and fixed at 1dpa (*i.e.* 3dpf, 1dpa), equivalent uncut controls (3dpf, uncut) were included. Embryos were subjected to whole-mount immunohistochemistry using a p-AKT-specific antibody (p-S473) (yellow). The embryos were counterstained with DAPI (blue). Representative images of embryo caudal fin-folds are shown, and in the left panels the edge of the fin-fold is indicated with a dashed line. Number of embryos showing similar patterns/ total number of embryos analysed is indicated in the bottom right corner. The scale bar represents 100µm. (D) p-AKT immunofluorescence was quantified by mean intensity of the region between the notochord and the edge of the caudal fin-fold. Means within amputated or uncut groups are compared to *ptena*$^{+/+}$*ptenb*$^{-/-}$ embryos. The number of embryos analysed is indicated (n). Significance: *** p<0.001; error bars represent standard deviation. Quantification of p-AKT immunofluorescence at 4dpf is depicted in Fig. 2B.

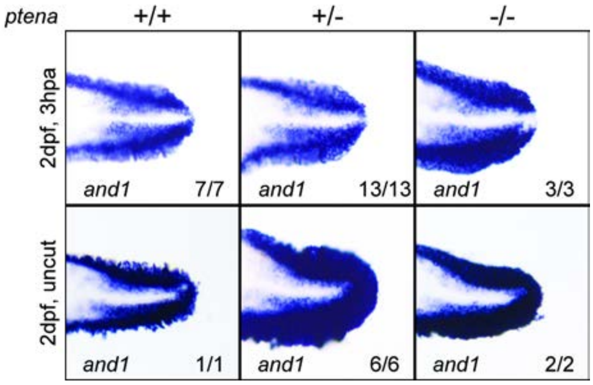

Fig. S4. *In situ* hybridization staining of *and1* in Pten deficient embryos. At 2dpf the caudal fin-fold of embryos from a *ptena*$^{+/-}$*ptenb*$^{-/-}$ in-cross was amputated and allowed to regenerate. Embryos were fixed at 3hpa, or equivalent for uncut controls, and subjected to *in situ* hybridization for *and1*. Representative images of embryo caudal fin-folds are shown with the number of embryos/ total number of embryos in the bottom right corner of each panel.

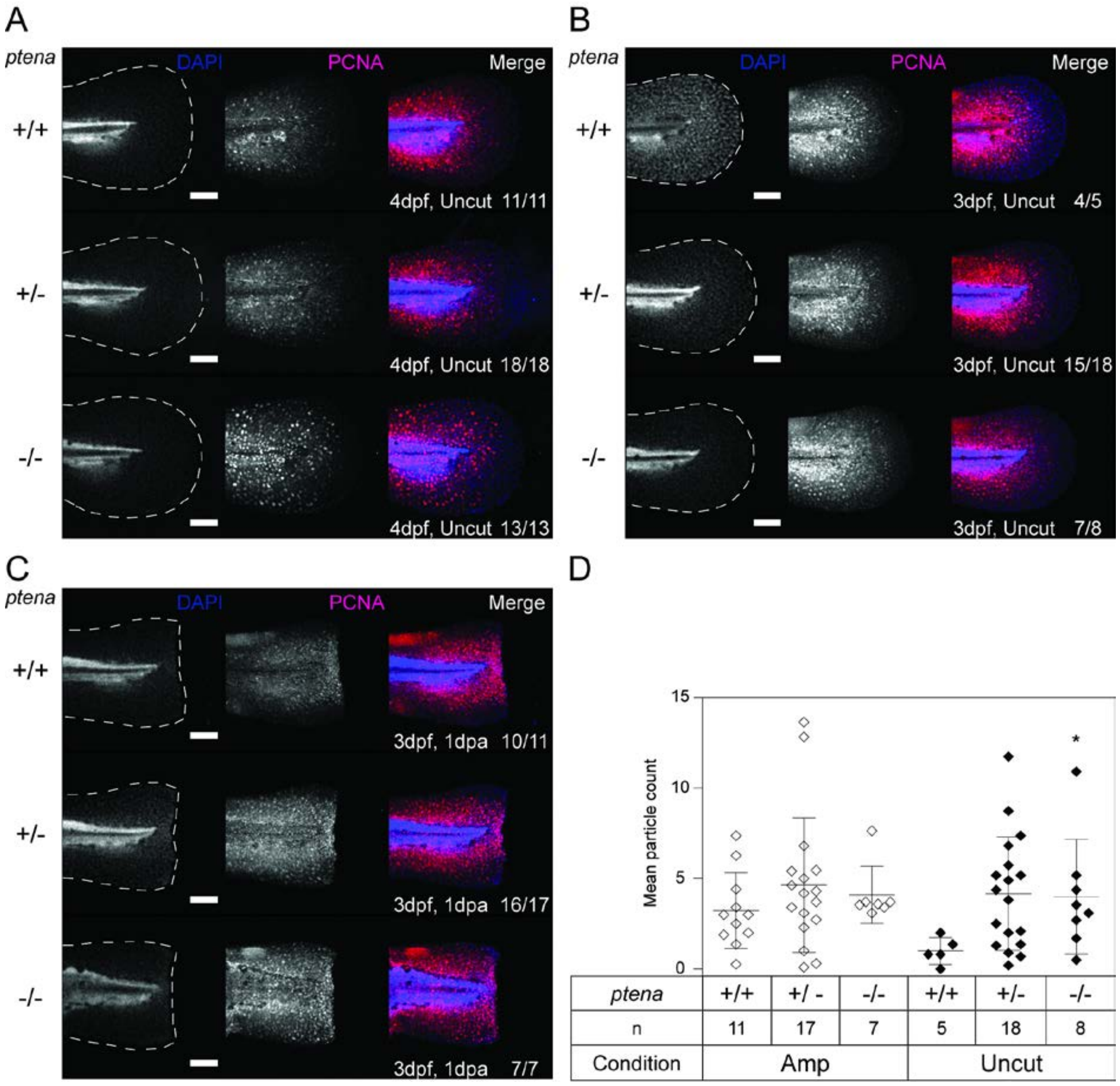

Fig. S5. Normal proliferation in the caudal fin-folds of Pten deficient embryos. (A) Uncut embryos from a *ptena*$^{+/-}$*ptenb*$^{-/-}$ in-cross were fixed at 4dpf (4dpf, uncut) in parallel to the embryos depicted in Fig. 5. (B, C) Embryos from a *ptena*$^{+/-}$*ptenb*$^{-/-}$ in-cross were amputated and fixed at 1dpa (*i.e.* 3dpf, 1dpa), equivalent uncut controls (3dpf, uncut) were included. Embryos were subjected to whole-mount immunohistochemistry using an antibody specific for the cell proliferation marker PCNA (red). The embryos were counterstained with DAPI (blue). Representative images of embryo caudal fin-folds are shown, and in the left panels the edge of the fin-fold is indicated with a dashed line. Number of embryos showing similar patterns/ total number of embryos analysed is indicated in the bottom right corner. The scale bar represents 100µm. (D) PCNA immunofluorescence between the tip of the notochord and edge of the caudal fin-fold at 3dpf was quantified by mean particle count, with thresholding and size restriction to remove background signal. Equivalent uncut controls were also quantified. Means within amputated or uncut groups were compared to *ptena*$^{+/+}$*ptenb*$^{-/-}$ embryos. Significance: * p<0.05; error bars represent standard deviation. Quantification of PCNA immunofluorescence at 4dpf is depicted in Fig. 6B.

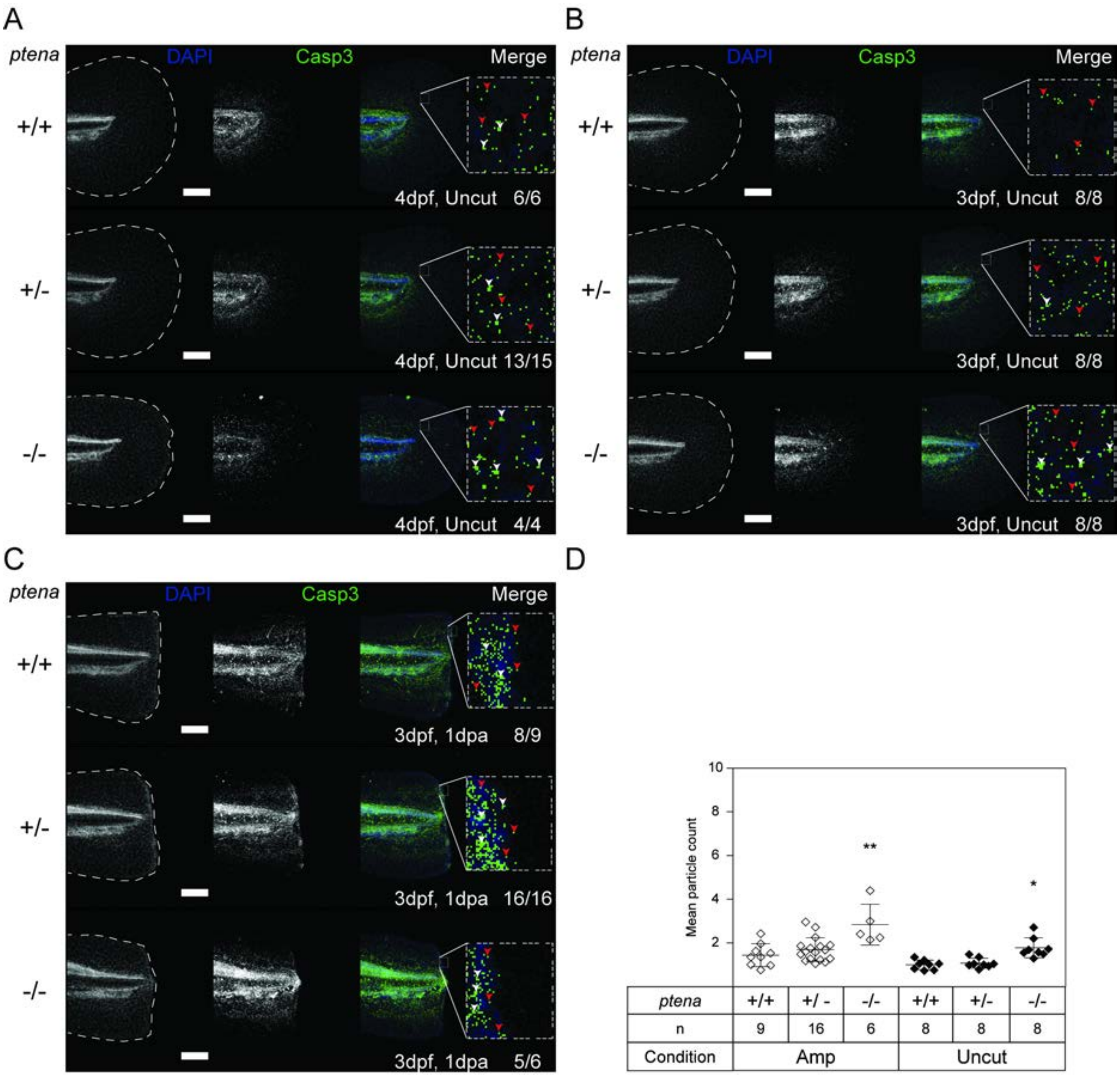

Fig. S6. Enhanced apoptosis in the caudal fin-folds of Pten deficient embryos. (A) Uncut embryos from a *ptena*$^{+/-}$*ptenb*$^{-/-}$ in-cross were fixed at 4dpf (4dpf, uncut) in parallel to the embryos depicted in Fig. 6A. (B, C) Embryos from a *ptena*$^{+/-}$*ptenb*$^{-/-}$ in-cross were amputated and fixed at 1dpa (*i.e.* 3dpf, 1dpa) , equivalent uncut controls (3dpf, uncut) were included. Embryos were subjected to whole-mount immunohistochemistry using an antibody specific for the apoptosis marker activated Caspase-3 (green). The embryos were counterstained with DAPI (blue). Representative images of embryo caudal fin-folds are shown, and in the left panels the edge of the fin-fold is indicated with a dashed line. Number of embryos showing similar patterns/ total number of embryos analysed is indicated in the bottom right corner. The scale bar represents 100µm. Zoom-in images of each caudal fin-fold are shown. White arrowheads indicate cells; red arrowheads indicate background staining. (D) Caspase-3 immunofluorescence was quantified by mean particle count of the caudal fin-fold following thresholding and size restriction to reduce background signal. Means within amputated or uncut groups are compared to *ptena*$^{+/+}$*ptenb*$^{-/-}$ embryos. The number of embryos analysed is indicated (n). Significance: * p<0.05; ** p<0.01; error bars represent standard deviation. Quantification of Caspase-3 immunofluorescence at 4dpf is depicted in Fig. 6B.

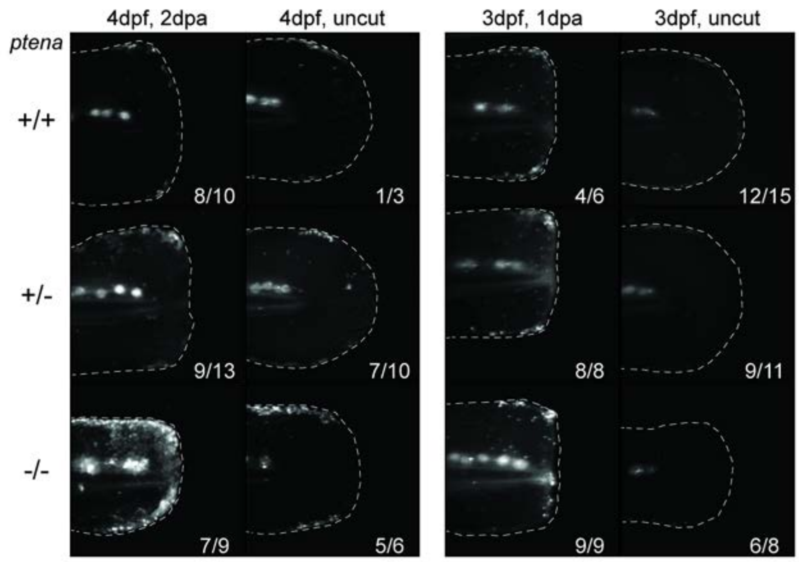

Fig. S7. Enhanced apoptosis in the caudal fin-folds of live Pten deficient embryos. Embryos from a *ptena*$^{+/-}$*ptenb*$^{-/-}$ in-cross were amputated and allowed to regenerate. Embryos were stained at 2dpa (*i.e.* 4dpf, 2dpa) or 1dpa (*i.e.* 3dpf, 1dpa), or equivalent for uncut controls (*i.e.* 4dpf, uncut; or 3dpf, uncut), with a dye for cells undergoing apoptosis, acridine orange, for 30mins. Representative images of embryo caudal fin-folds at are shown, and the edge of the fin-fold is indicated with a dashed line. Number of embryos showing similar patterns/ total number of embryos analysed is indicated in the bottom right corner. Representative images for embryos stained at 4dpf, 2dpa are shown in Fig. 7C.
